# Antioxidant Activity of Hispidin Oligomers from Medicinal Fungi: A DFT Study

**DOI:** 10.3390/molecules19033489

**Published:** 2014-03-21

**Authors:** El Hassane Anouar, Syed Adnan Ali Shah, Normahanim Binti Hassan, Najoua El Moussaoui, Rohaya Ahmad, Mohd Zulkefeli, Jean-Frédéric F. Weber

**Affiliations:** 1 Atta-ur-Rahman Institute for Natural Products Discovery, Level 9, FF3, Universiti Teknologi MARA, Puncak Alam Campus, 42300 Bandar Puncak Alam, Selangor Darul Ehsan, Malaysia; 2 Faculty of Pharmacy, Universiti Teknologi MARA (UiTM), Puncak Alam Campus, 42300 Bandar Puncak Alam, Selangor Darul Ehsan, Malaysia; 3 Department of Biology, Science Faculty, Abdelmalek Essaâdi University, Tetouan, 2121, Morocco; 4 Faculty of Applied Sciences, Universiti Teknologi MARA, 40450 Shah Alam, Selangor Darul Ehsan, Malaysia

**Keywords:** hispidin, antioxidant activity, DFT, BDE, PC-ET

## Abstract

Hispidin oligomers are styrylpyrone pigments isolated from the medicinal fungi *Inonotus xeranticus* and *Phellinus linteus*. They exhibit diverse biological activities and strong free radical scavenging activity. To rationalize the antioxidant activity of a series of four hispidin oligomers and determine the favored mechanism involved in free radical scavenging, DFT calculations were carried out at the B3P86/6-31+G (d, p) level of theory in gas and solvent. The results showed that bond dissociation enthalpies of OH groups of hispidin oligomers (ArOH) and spin density delocalization of related radicals (ArO^•^) are the appropriate parameters to clarify the differences between the observed antioxidant activities for the four oligomers. The effect of the number of hydroxyl groups and presence of a catechol moiety conjugated to a double bond on the antioxidant activity were determined. Thermodynamic and kinetic studies showed that the PC-ET mechanism is the main mechanism involved in free radical scavenging. The spin density distribution over phenoxyl radicals allows a better understanding of the hispidin oligomers formation.

## 1. Introduction

**Figure 1:**
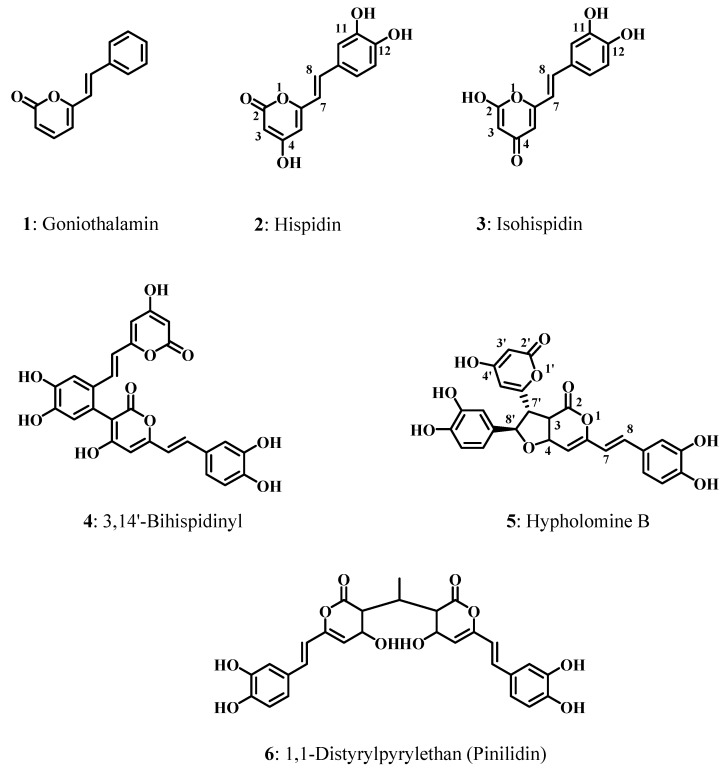
Molecular structures of compounds **1–6**.

Styrylpyrones form a small group of natural phenolics of which goniothalamin (**1**, Figure 1), a cytotoxic plant derivative, is the best known representative [1]. A fascinating series of styrylpyrone oligomers and adducts was isolated from mushrooms belonging to the *Inonotus* and *Phellinus* genera (Hymenochaetaceae family), with hispidin (**2**, Figure 1) as a prototype [2]. The key structural difference with goniothalamin-type compounds lies in the presence of a highly oxidisable 4-hydroxy group, which can be held responsible for the reactivity of hispidin and its derivatives. These lignicolous mushrooms are traditionally used in Russia and western Siberia as folk medicines for the treatment of various serious diseases including cancer, stomach, liver or heart diseases [3,4,5]. Most of these diseases are related to oxidative stress, which results from the overproduction of free radicals and specifically reactive oxygen species [6].

Experimental and theoretical studies of the structure-antioxidant activity relationships of natural polyphenols proved that this activity mainly depends on phenolic hydroxyl groups, and their capacity to donate a hydrogen atom to quench free radicals. In addition to the experimental studies on the quantitative structure-antioxidant activity relationship (QSAR), quantum chemical calculations showed to be a potent tool to rationalize structure-antioxidant activity of polyphenolic compounds [7,8,9,10,11,12,13,14,15,16]. Density functional theory (DFT) has become a very popular tool to rationalize QSAR, since it successfully describes the hydrogen atom transfer of OH phenolic groups to quench free radicals. Several structural features are known to enhance the antioxidant activity. Among them are (i) the presence of a catechol moiety, which strongly participates in the free radical scavenging capacity of flavonoids [17], oligomers of guaiacol [11], chalcones [10], hydroxybenzohydroxamic acid derivatives [18], as well as hispidin oligomers [19]; and (ii) 3-OH and C2=C3 double bond in flavonoids [12,17].

In the present paper, we report the study of the structure-antioxidant activity relationships of a series of hispidin oligomers using DFT calculations. A series of five hispidin oligomers were isolated from culture broths of *I. xeranticus* and *P. linteus* [19]. They include hispidin (**2**), isohispidin (**3**), 3,14'-bihispidinyl (**4**), hypholomin B (**5**) and 1,1-distyrylpyrylethan (**6**) (Figure 1). Compounds **3–5** are believed to be biosynthesized by dimerization of hispidin. Their antioxidant activity or, more specifically, ability to scavenge DPPH (1,1-diphenyl-2-picrylhydrazyl) free radical was determined by Jung *et al.* [19] through Blois’ method [20]. 

## 2. Results and Discussion

### 2.1. Hispidin Dimerization

**Figure 2:**
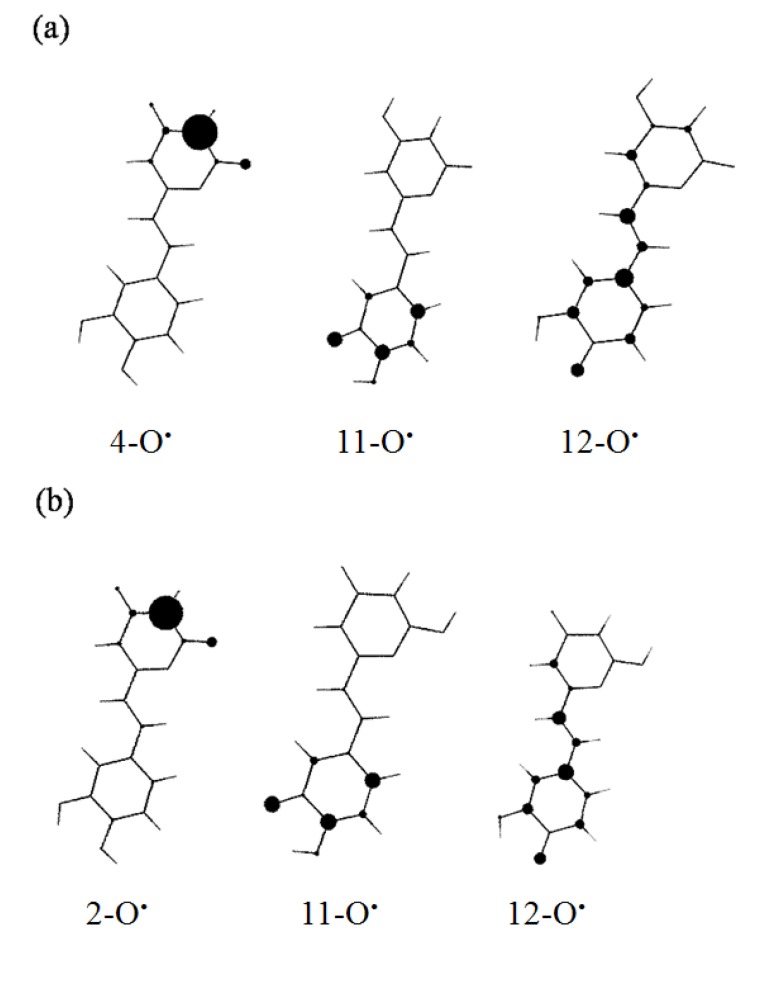
Electronic spin density distribution of i-O^•^ hispidinyl and isohispidinyl radicals (**a**) 4-O^•^, 11-O^•^ and 12-O^•^ hispidinyl radicals; (**b**) 2-O^•^, 11-O^•^ and 12-O^•^ isohispidinyl radicals.

Various styrylpyrone metabolites can be biogenerated by dimerization or oligomerization process [21]. For instance, hispidin oligomers **4** and **5** are likely to derive from dimerization of hispidin. The dimerization of hispidin is believed to proceed through a free-radical chain reaction. In the initiation step, the oxidation of hispidin leads to the formation of an unstable ArOH^+•^ radical cation. This is followed by a proton loss (heterolytic rupture of the OH bond of ArOH^+•^), thus forming a hispidinyl ArO^•^ radical. The hispidinyl and isohispidinyl radicals display a strong electronic spin density on oxygen and few carbon atoms (Figure 2). During the propagation and termination steps, the coupling between hispidinyl and isohispidinyl radicals generates various dimers. For instance, the coupling between the C3 of 4-O^•^ free radical and C13 of the 11-O^•^ hispidinyl radicals leads to the formation of dimer **4**.

### 2.2. Structure-Antioxidant Activity Relationships

#### 2.2.1. Tautomerism Effect

**Figure 3:**
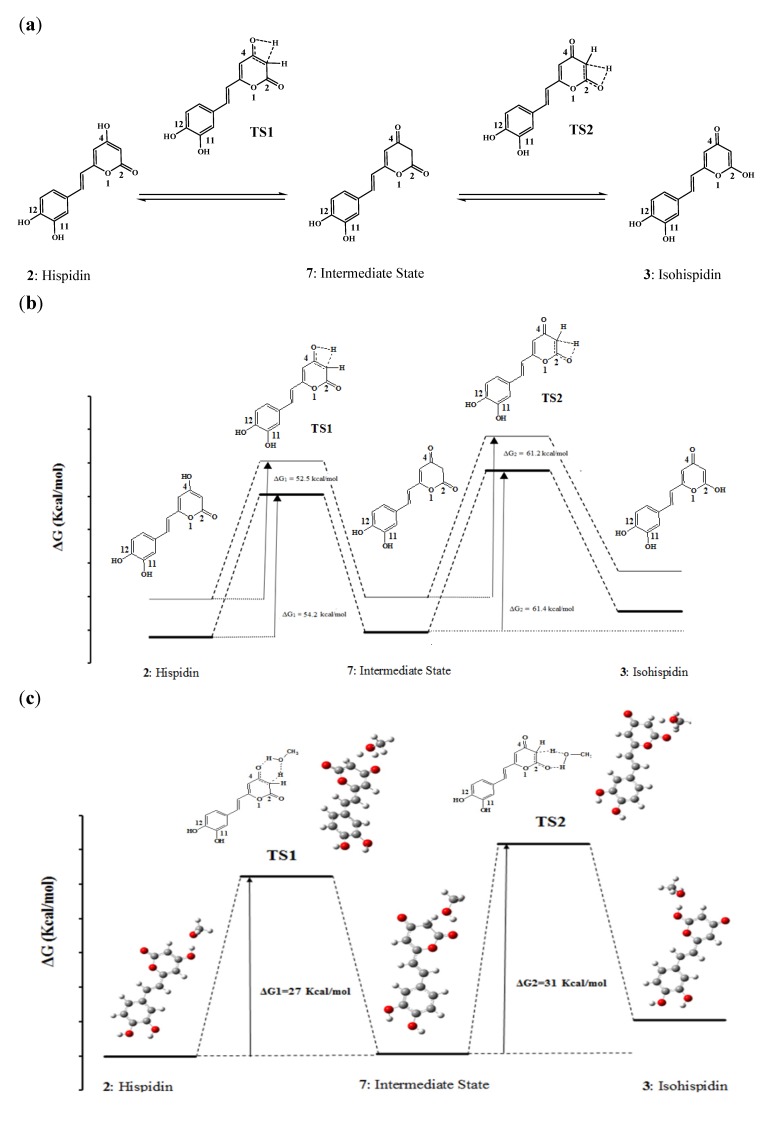
(**a**) Tautomerism of hispidin to isohispidin; (**b**) tautomerism chemical profiles in gas and solvent; and (**c**) tautomerism chemical profile in hybrid model (PCM + one assisted solvent molecule).

The tautomerization of hispidin (**2**) leads to the formation of stable isohispidin (**3**, Figure 3a). The equilibrium is a two-step reaction, with 4-oxohispidin (**7**) as intermediate state. Two transition states were determined in the gas phase and in PCM at B3P86/6-31+G (d, p) levels. As can be seen in the coordinate reaction diagrams (Figure 3b,c), the initial compound hispidin is more stable than the final isohispidin. The equilibrium shifts toward the lower Gibbs energy and the formation of hispidin should be thus favored. However, the presence of two transition states on both sides of intermediate state **7** indicates that the intermediate must overcome an activation energy barrier before reverting to **2** or **3**. The activation energies calculated in gas between hispidin and TS1, and between the intermediate and TS2 are 52 and 61 kcal/mol, respectively. PCM has negligible effects on activation energies with a variation of less than 2 kcal/mol. The high activation barriers can be related to the one-step direct intramolecular hydrogen transfer mechanisms that take place through highly strained four-membered transition states. An alternative mechanism that would explicitly involve a solvent molecule would allow considering an indirect hydrogen transfer through a six-membered cycle transition state that would be far less strained and thus significantly decrease the energy barriers. Such an approach has been employed in several studies. Emamian *et al.* studied the solvent effect on the tautomerization of pyridazinone into pyridazole by combining explicit and solvation models and highlighted the significance of explicit solvent molecules involved in six or higher membered TSs in reducing the activation barriers [22]. Chahkandi *et al.* investigated the effect of 1-3 water assisted molecules on the activation energies of the tautomerism reaction of 1,3,4-oxadiazole derivatives and found that the energy barriers were strongly reduced in presence of water molecules [23]. We thus used an hybrid model *i.e.*, PCM + one solvent molecule (Figure 3c). Taking into account an assisting solvent molecule results in a significant decrease of the energy barriers to 27 and 31 kcal/mol for TS1 and TS2, respectively. This shows that an intramolecular mechanism is much less likely than a solvent assisted mechanism.

From these results, it appears that hispidin is more stable compared to isohispidin and that interconversion is unlikely at room temperature, thus explaining the description of hispidin and isohispidin as two distinct compounds.

BDEs were calculated for both tautomers in order to study possible differences in antioxidant activity. The results showed similar BDEs for both tautomers (Table 1). For instance, the BDE difference between the active OH groups in hispidin and isohispidin is less than 0.2 kcal/mol and 0.3 kcal/mol in the gas phase and PCM, respectively.

**Table 1:** BDEs and IPs of hispidin and isohispidin calculated using the B3P86 hybrid functionals and 6-31+G(d,p) and 6-311+G(d,p) basis sets.

Compounds	Gas		PCM	
	B3P86/6-31+G(d,p)	B3P86/6-311+G(d,p)	B3P86/6-31+G(d,p)	B3P86/6-311+G(d,p)
	BDE (kcal/mol)			
Hispidin				
4-OH	90.0	88.2	90.6	89.5
11-OH	78.9	78.7	80.3	80.0
12-OH	75.0	74.6	76.2	75.9
Isohispidin				
2-OH	79.2	79.0	80.9	80.6
11-OH	79.0	78.9	80.3	80.0
12-OH	75.2	74.9	76.5	76.1
	**IP(eV)**			
Hispidin	7.75	7.82	6.24	6.30
Isohispidin	7.96	8.10	6.39	6.39

#### 2.2.2. Isomerism Effect

**Figure 4:**
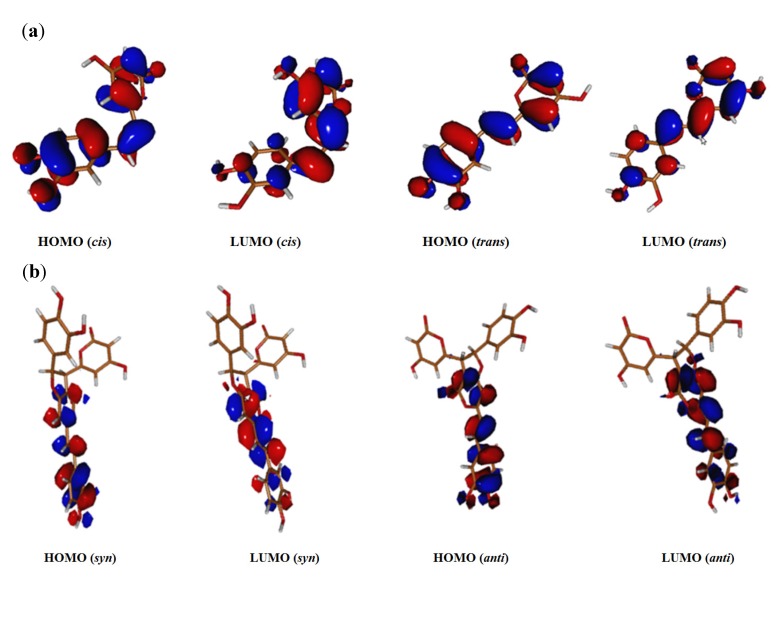
HOMO and LUMO distributions for styrylpyrones (**a**) *cis* and *trans* hispidin (**2**) isomers; (**b**) *syn* and *anti* hypholomine B (**5**) isomers.

Styrylpyrone oligomers often exist in isomeric and conformer forms. We had previously tested the conformation effect on the antioxidant activity of a series of guaiacol oligomers and showed that the stable conformers have similar BDEs, IPs, HOMO and LUMO molecular orbitals parameters [11]. For hispidin oligomers, due to the restricted rotation of C7=C8, only *cis* and *trans* isomers need to be considered. We calculated BDEs, IPs and HOMO and LUMO distribution for *cis* and *trans* hispidin isomers (Table 2 and Figure 4a). Similar BDEs values were obtained for both isomers (differences less than 0.6 kcal/mol in solvent) and IP (7.3 eV). The HOMO and LUMO molecular orbitals were also found very similar for both *cis* and *trans* isomers (Figure 4). The *trans* isomer is more stable than *cis* isomer by 9 kcal/mol. In *trans* isomer, the structure is totally planar, while the structure of the *cis* isomer slightly deviates out of plane due to the steric hindrance between the aromatic and lactone rings. However, conjugation over the entire molecule is observed for both *cis* and *trans* isomers (Figure 4a). 

**Table 2:** BDEs and IPs of hispidin oligomers calculated at B3P86/6-31+G(d,p) level.

Compounds	BDEs (kcal/mol)						IP (eV)	IC_50_ (µmol/L)
	4-OH	11-OH	12-OH	4'-OH	11'-OH	12'-OH		
**(a) Gas phase**								
Hispidin (*trans*)	90.0	78.9	75.0	-	-	-	7.75	1.31 ± 0.81
Hispidin (*cis*)	89.1	78.2	76.0	-	-	-	7.95	1.31 ± 0.81
3,14'-Bihispidinyl	83.6	78.9	74.8	89.2	77.1	74.6	7.08	0.90 ± 0.61
Hypholomine B (*syn*)	-	77.4	73.9	89.9	77.5	77.5	7.7	0.31 ± 0.22
Hypholomine B (*anti*)	-	88.6	75.4	91.2	77.8	77.7	7.7	0.31 ± 0.22
1,1-Distyrylpyrylethan	101.2	78.8	74.6	99.2	88.6	74.7	7.2	0.37 ± 0.15
**(b) PCM**								
Hispidin (*trans*)	90.6	80.3	76.2	-	-	-	6.24	1.31 ± 0.81
Hispidin (*cis*)	90.9	79.3	77.0	-	-	-	6.30	1.31 ± 0.81
3,14'-Bihispidinyl	84.8	80.4	76.3	90.2	79.3	76.6	6.10	0.90 ± 0.61
Hypholomine B (*syn*)	-	79.1	75.7	92.8	79.4	79.2	6.3	0.31 ± 0.22
Hypholomine B (*anti*)	-	84.7	76.3	92.7	79.9	79.7	6.3	0.31 ± 0.22
1,1-Distyrylpyrylethan	97.2	79.7	76.1	94.4	85.4	76.2	6.2	0.37 ± 0.15

In case of dimer **5**, the orientation of the lactone and aromatic rings with respect to the covalent bond (C7'-C8') in the tetrahydrofuran ring leads to *syn-* and *anti-*isomers. Recently, we showed that *syn* and *anti* terrein isomers displayed similar antioxidant activities [24]. Urbaniak *et al.* showed that *E* and *Z* ferulic acid have similar free radical scavenging capacity [25]. In accordance with hispidin isomers results, both *syn-* and *anti*-dimer **5** isomers showed similar theoretical results (Table 2 and Figure 4b). For the other hispidin oligomers we only considered the *anti*-isomers, as they were identified experimentally as the stable isomers. For dimers **4** and **6**, the free rotation of the C-C covalent bonds (linking hispidin moieties) gives rise to diverse conformers. Two conformers were obtained for **4** with torsion angles of 60 and 120 degrees. For these two conformers, the hydrogen bonds between (i) OH groups and (ii) OH groups and carbonyl group were taken into consideration. Calculations showed that the conformation had little influence on theoretical parameters. Therefore, only the most stable conformer with the lowest energy and intramolecular hydrogen bonds was considered.

#### 2.2.3. BDEs Analysis

**Figure 5:**
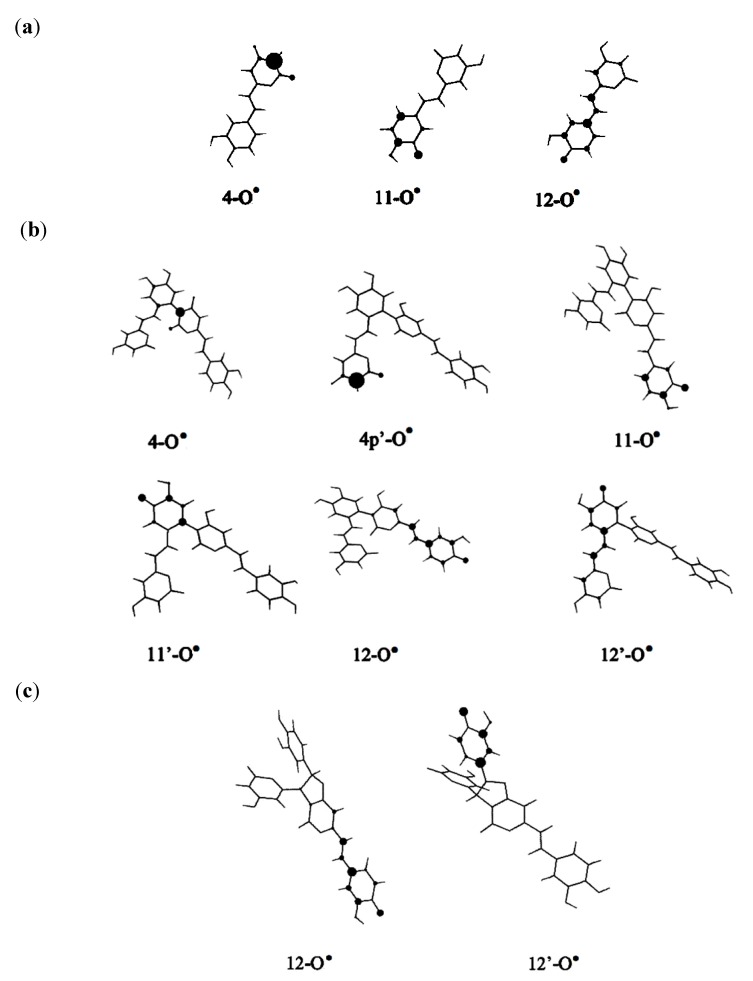
Spin density distribution for i-O^●^ styrylpyrone radicals (**a**) hispidin; (**b**) 3,14'-bihispidinyl; (**c**) hypholomine B.

On the basis of IC_50_ (the capacity of hispidin oligomer to scavenge 50% of DPPH free radical) values (Table 2), hispidin oligomers were capable of scavenging DPPH free radical in the following order: hypholomine B > 1,1-distyrylpyrylethane > 3,14'-bihispidinyl > hispidin [19]. For each hispidin oligomer, the lowest BDEs were obtained for 12-OH and 12'-OH groups of catechol rings (Table 2). The BDEs values are relatively close to each other, with differences less than 0.6 kcal/mol in PCM. Such differences do not explain the observed results. The lowest BDEs being associated with 12-OH groups come from the fact that the electronic spin density distribution of 12-OH phenoxyl radical is delocalized over the radical, which is not the case for other i-OH radicals (Figure 5). The high antioxidant activity of dimers **4–6** with regard to hispidin is probably related to the number of catechol and OH groups (Figure 1). This result was consistent with the good antioxidant role of the catechol moiety generally observed for polyphenols. The BDEs values of hispidin oligomers are relatively similar, thus the correlation to experiment results is comparatively weak. To improve such correlation, we calculated BDE_d_ parameter (BDEs calculated after a second hydrogen atom transfer), which showed a good correlation to the antioxidant activity of guaiacol oligomers [11]. 

#### 2.2.4. Double BDEs Analysis

In Table 3, we report BDE_d_ for OH groups (after a second hydrogen atom transfer) and IP_d_ for hispidin oligomers in the gas phase and PCM. For each hispidin oligomers, the lowest BDE_d_ is obtained for the 11-OH group. The lowest of all BDE_d_ is obtained for the 11-OH group of **5** with a value of 77.4 kcal/mol. This BDE_d_ is in a good agreement with the experimental results, which showed that oligomer **5** is the most potent antioxidant among tested hispidin oligomers with the lowest IC_50_ value (Table 3). The BDE_d_ of 11-OH groups of **4** and **5** are relatively similar (Table 2). The high antioxidant activity of **5** with respect to **4** could be explained by the low BDE of a third hydrogen atom transfer (BDE_t_). These results are in agreement with recent results reported by Amić *et al.* who uncovered the significant role of BDE_d_ and IP_d_ for a double PCET mechanism of free radical scavenging potency in flavonoids [26].

**Table 3:** BDE_d_ and IP_d_ of hispidin oligomers calculated at B3P86/6-31+G(d,p) level.

Compounds	BDE_d_ (kcal/mol)					IP_d_ (eV)	IC_50_ (µmol/L)
	4-OH	11-OH	4'-OH	11'-OH	12'-OH		
**(a) Gas phase**							
Hispidin	99.3	81.8	-	-	-	7.9	1.31 ± 0.81
3,14'-Bihispidinyl	89.4	82.2	106.9	82.1	87.6	7.4	0.90 ± 0.61
Hypholomine B ( *anti*)	-	81.2	111.0	96.2	95.7	7.9	0.31 ± 0.22
1,1-Distyrylpyrylethan	108.4	81.7	100.8	97.2	90.6	7.4	0.37 ± 0.15
**(b) PCM**							
Hispidin	93.9	78.2	-	-	-	6.3	1.31 ± 0.81
3,14'-Bihispidinyl	89.4	78.9	99.0	84.5	89.0	6.2	0.90 ± 0.61
Hypholomine B ( *anti*)	-	77.4	101.1	97.6	97.0	6.3	0.31 ± 0.22
1,1-Distyrylpyrylethan	101.3	78.8	98.5	97.5	91.2	6.2	0.37 ± 0.15

#### 2.2.5. Effect of the Number of OH Groups and Conjugation of the Catechol Moiety

QSAR studies of antioxidant activity of polyphenols confirmed the significant role of the number of hydroxyl groups and catechol moieties conjugated to double bond in increasing the antioxidant activity [17,27]. These effects are well illustrated on the present series of hispidin derivatives. For instance, hispidin **2**, with three hydroxyl groups, is less active than its dimers **4** and **5** with five and six hydroxyl groups respectively. Hypholomine B (**5**) possesses two catechol moieties; on one side the catechol moiety is conjugated to a double bond (C7=C8) and on the other side the catechol is attached to a simple covalent bond (C7'-C8'). The difference between BDEs values of 12'-OH and 12-OH is 2.3 kcal/mol. This difference demonstrates the importance of a double bond conjugated to a catechol moiety, which extends the electronic spin delocalization over the radical (Figure 5c). The improvement of the antioxidant activity of dimer **4** compared to hispidin **2** can be related to the elongation of the conjugated chain. This result is in good agreement with a recent study by Lu *et al.* who showed that the elongation of resveratrol chain significantly increases the antioxidant activity [28]. 

### 2.3. Thermodynamical and Kinetic Study

#### 2.3.1. Thermodynamic of the PC-ET Mechanism

**Figure 6:**
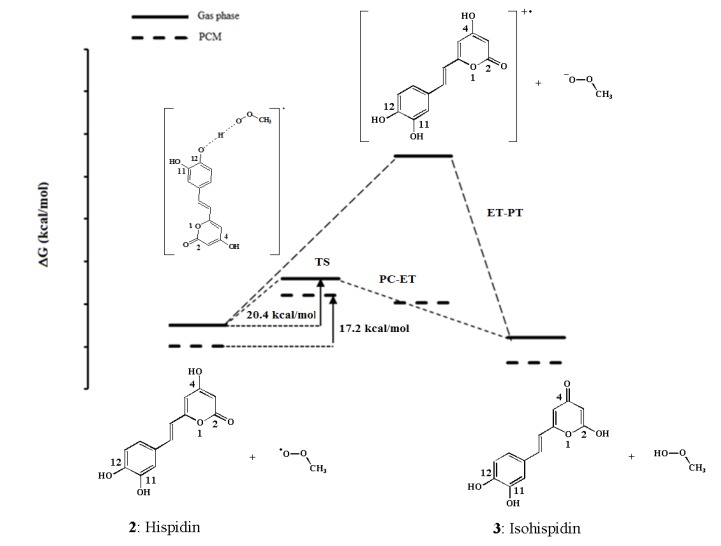
Chemical profiles for PC-ET and ET-PT mechanisms.

To correlate theoretical parameters and experimental results for DPPH free radical scavenging activity, free Gibbs energies (ΔG) were evaluated for the reaction between hispidin oligomers and DPPH or CH_3_OO^•^ free radicals. For both free radicals, the PC-ET and ET-PT mechanisms were studied (Figure 6 and Table 4). ΔG^ET^ values of the first step of the ET-PT mechanism are strongly influenced by the PCM [10,11], where the solvent induces a decrease of 55–70 and 90–116 kcal/mol for DPPH and CH_3_OO^•^, respectively. Even in PCM, the ET mechanism is endergonic. It must be stressed that the ET physical process is just a first step that is followed by heterolytic bond dissociation. ΔG^ET−PT^ results from these two steps. The reactions between hispidin oligomers and DPPH radical are exergonic with ΔG^PC−ET^ close to −4 kcal/mol (Table 5a). 

**Table 4:** Thermodynamics (ΔG^PC−ET^ and ΔG^ET^ in kcal/mol) of the DPPH and CH_3_OO^•^ free radical scavenging by styrylpyrone derivatives.

	ΔG^PC−ET^				ΔG^ET^			
	DPPH		CH_3_OO^•^		DPPH		CH_3_OO^•^	
	Gas	PCM	Gas	PCM	Gas	PCM	Gas	PCM
**(a) B3P86//First PC-ET**								
Hispidin	−4.27	−0.12	−8.76	−8.13	88.50	22.20	143.63	41.55
3,14'−Bihispidinyl	−4.10	0.09	−8.59	−7.91	74.25	19.45	129.38	38.80
Hypholomine B	−3.29	−0.28	−7.78	−8.28	87.50	22.63	142.63	41.98
Pinilidin	−4.54	−0.15	−9.02	−8.16	75.65	20.21	130.78	39.56
**(b) B3P86//Second PC-ET**								
Hispidin	4.04	3.42	−0.45	−4.58	93.84	25.88	148.97	45.22
3,14'-Bihispidinyl	2.41	2.06	−2.07	−5.94	81.36	22.98	136.49	42.32
Hypholomine B	1.67	1.13	−2.82	−6.88	92.45	25.64	147.59	44.99
Pinilidin	4.09	2.04	−0.40	−5.97	82.16	23.00	137.29	42.34
**(c) MPWB1K//First PC-ET**								
Hispidin	−2.26	1.34	−5.78	−5.07	43.94	26.24	163.06	46.97
3,14'-Bihispidinyl	−2.00	1.51	−5.33	−4.90	81.13	24.85	146.25	45.58
Hypholomine B	−1.18	1.22	−4.71	−5.19	97.51	27.24	162.63	47.96
Pinilidin	−2.36	1.39	−5.89	−4.41	85.23	25.53	150.35	123.55
**(d) MPWB1K//Second PC-ET**								
Hispidin	18.25	17.15	0.13	−3.66	109.41	40.95	163.91	50.20
3,14'-Bihispidinyl	16.92	15.79	13.10	9.38	96.93	38.05	162.05	50.87
Hypholomine B	15.88	14.85	12.35	8.44	108.2	40.72	173.14	61.44
Pinilidin	18.30	15.76	14.77	9.35	97.73	38.07	162.85	58.79

ΔG^PC−ET^ values for a second PC-ET are shown in Table 4b. In case of peroxyl radical scavenging by hispidin oligomers, the second PC-ET is thermodynamically favored with a ΔG^PC−ET^ close to −6 kcal/mol.

#### 2.3.2. Kinetics of the Reaction Styrylpyrone + CH3OO• → [styrylpyrone-H]• + CH3OOH

According to the thermodynamic data of ET-PT, the first step was clearly the limiting step (e.g., for the hispidin reactivity with CH_3_OO^•^, ΔG^ET^ = +41.55 kcal/mol and ΔG^PT^ = −49.68 kcal/mol in PCM. The ET transition states cannot be calculated exactly, but the activation free energy is obviously higher than ΔG^ET^ (*i.e.*, higher than 41.55 kcal/mol). The transition state is close to the energy required for a vertical transition, which was calculated to be at least 3–4 kcal/mol higher than ΔG^ET^. Thus, the energy of activation would be significantly higher for ET than for the first PC-ET mechanism, which makes the ET rates higher than those of PC-ET. This indicates that PC-ET was faster than ET-PT. PC-ET appears as a major mechanism, while ET-PT would be minor for this series of compounds. The relatively small barriers (or high rate constants) obtained for the major mechanism (PC-ET) seem to indicate that the reaction is mainly thermodynamically governed (Table 5). It is known that the hybrid functional B3P86 underestimates activation barriers. Indeed, our results appear as too low. Better results were obtained with hybrid functional MBWB1K (Table 5). 

**Table 5:** Calculated thermodynamics (ΔG in kcal/mol) and kinetics (ΔG^#^ in kcal/mol and K in M^−1^·s^−1^) parameters of PC-ET mechanism of the CH3OO^•^ (R^•^) free radical scavenging by styrylpyrones.

	ΔG	ΔG^#^	K^TST^	K^TST/W^	K^TST/ST^
(**a) B3P86//Gas phase**					
Hispidin + R^•^ → [Hispidin-H]^•^ + RH	−8.76	4.89	1.61*10^9^	4.29*10^9^	1.59*10^10^
3,14'-Bihispidinyl + R^•^ → [3,14'-Bihispidinyl -H]^• ^+ RH	−8.59	5.15	1.04*10^9^	2.73*10^9^	3.63*10^9^
Hypholomine B + R^•^ → [Hypholomine B -H]^•^ + RH	−7.78	6.00	2.49*10^9^	6.93*10^8^	2.74*10^9^
Pinilidin + R^•^ → [Pinilidin -H]^•^ + RH	−9.02	4.89	1.61*10^9^	4.08*10^9^	1.28*10^10^
**(b) B3P86//PCM**					
Hispidin + R^•^ → [Hispidin-H]^•^ + RH	−8.13	8.55	3.32*10^6^	1.52*10^7^	1.20*10^7^
3,14'-Bihispidinyl + R^•^ → [3,14'-Bihispidinyl -H]^•^ + RH	−7.91	11.30	3.20*10^4^	1.62*10^5^	1.02*10^5^
Hypholomine B + R^•^ → [Hypholomine B -H]^•^ + RH	−8.28	8.22	5.87*10^6^	2.66*10^7^	1.84*10^7^
Pinilidin + R^•^ → [Pinilidin -H]^•^ + RH	−8.16	5.66	1.62*10^9^	1.28*10^9^	1.80*10^10^
**(c) MPWB1K//Gas phase**					
Hispidin + R^•^ → [Hispidin-H]^•^ + RH	−5.78	16.96	2.36	6.30	2.68*10^1^
3,14'-Bihispidinyl + R^•^ → [3,14'-Bihispidinyl -H]^•^ + RH	−5.33	17.20	1.58	4.12	7.35
Hypholomine B + R^•^ → [Hypholomine B -H]^•^ + RH	−4.71	18.02	3.96*10^−1^	1.10	5.66
Pinilidin + R^•^ → [Pinilidin -H]^•^ + RH	−5.89	17.24	1.47	3.73	13.31
**(d) MPWB1K//PCM**					
Hispidin + R^•^ → [Hispidin-H]^•^ + RH	−5.07	17.64	7.40*10^−1^	3.38	1.47*10^2^
3,14'-Bihispidinyl + R^•^ → [3,14'-Bihispidinyl -H]^•^ + RH	−4.9	20.40	7.03*10^−3^	3.56*10^−2^	2.41
Hypholomine B + R^•^ → [Hypholomine B -H]^•^ + RH	−5.19	17.82	5.46*10^−1^	2.48	1.49*10^2^
Pinilidin + R^•^ → [Pinilidin -H]^•^ + RH	−4.41	16.02	1.14*10^1^	5.10*10^1^	3.51*10^3^

These results highlight the importance of thermodynamic parameters (BDE, BDE_D_, and ΔG^PC−ET^) to rationalize the antioxidant activity of hispidin oligomers. In addition to BDE and BDE_d_ parameters, the abstraction of the hydrogen atom depends on steric hindrance and electrostatic interaction between the ArOH and ^•^OOCH_3_ in the transition states. The activation energies decrease with the increase of electrostatic interactions. In the current study, the activation energy was found inversely proportional to d_H…OOCH3_ (R^2^ = 72%) and proportional to d_ArO…H_ (R^2^ = 69%) in the transition states.

## 3. Methodology

It is well known that polyphenols ArOH (e.g., styrylpyrones) scavenge free radicals (R^•^) by their ability to donate hydrogen atoms from hydroxyl groups via the following probable mechanisms:

(i) *Proton Coupled-Electron Transfer (PC-ET) versus Hydrogen atom transfer (HAT)*
ArOH + R^•^ → ArO^•^ + RH
  In the above reaction, the electron and proton are transferred from the active phenolic hydroxyl group to the free radical in a single step. This type of reactions can be subdivided into two distinct subclasses, hydrogen atom transfer (HAT) and proton coupled electron transfer (PC-ET) [14,29,30]. In HAT, the proton and electron are transferred together, as a hydrogen atom. In PC-ET mechanism, the proton and electron are transferred between different sets of orbitals [14]. Depending on the structure of reactants and the environment (e.g., solvent, enzyme), this reaction can proceed via HAT or PC-ET [31]. Various criteria can be used to distinguish between HAT and PC-ET. Tishchenko *et al.* used electronic adiabacity and nonadiabactity criterion to determine the mechanism involved in the reactivity of phenol with ^•^NH_2_ and ^•^OOCH_3_ radicals [31]. With regard to this criterion, HAT is electronically adiabatic, while PC-ET involves electronic nonadiabatic effects [31]. By using orbital-based criterion, Mayer *et al.* found that the degenerate hydrogen atom exchange reaction between phenoxyl radical (PhO^•^) and phenol (PhOH) involves a transfer of a proton and an electron between two different sets of molecular orbitals (PC-ET mechanism), while the reaction between benzyl radical and toluene involves the transfer of proton and electron between the same sets of molecular orbitals (HAT mechanism) [14]. As shown by Mayer *et al.* and Oksana *et al.* the reactivity of phenols with free radicals such CH_3_OO^•^ and PhO^•^ involve a PC-ET mechanism [14,31]. Therefore, one can consider that the reactivity of the current series of hispidin oligomers (ArOH) with CH_3_OO^•^ proceeds through a PC-ET mechanism. The natural bond orbitals (NBO) charges were calculated for the reactants (hispidin oligomers and peroxy radical) and TS structures using both hybrid functionals B3P86 and MPWB1K in gas and solvent. The results showed a slight variation of NBO charges of the abstracted hydrogen atom between the TS and the reactants. For instance, a slight increase of 0.02 was obtained in gas phase with MPWB1K hybrid functional. However, the sum of the oxygen atoms (bold atoms) in the reactants [Ph-**O**-H + ^•^**O**-OCH_3_] and transition state [Ph-**O**…H…**O**-OCH_3_]^•^ are −0.90 and −1.06, respectively. Thus, −0.16 more negative charge resides on the oxygen at the TS than in the reactants. In the PC-ET mechanism of phenoxyl/phenol, Mayer *et al.* found −0.17 more negative charge residing on oxygen atoms at the TS than in the reactants [14]. From this results, the reactivity of the hispidin oligomers with CH_3_OO^•^ can be considered as proceeding through a PC-ET mechanism.
  The O-H bond dissociation is homolytic. Therefore, the bond dissociation enthalpy (BDE) of OH groups is considered as the main parameter that governs this mechanism. BDEs are calculated for each OH group of hispidin oligomers by the following formula:
BDE = H(ArO^•^, 298K) + H(H^•^, 298K) − H(ArOH, 298K), (1)
  where H is the enthalpy that takes into account temperature-dependent corrections [zero point energy (ZPE), translational, rotational and vibrational energies at 298K]; H (ArOH, 298K) and H (ArO^•^, 298K) are the enthalpies of the hispidin oligomer and its corresponding radical (obtained after homolytic OH bond dissociation), respectively; and H (H^•^, 298K) is the enthalpy of hydrogen radical. BDE is an intrinsic parameter that helps to estimate the capacity of a compound to lose an hydrogen atom. The lower is the BDE, the easier is the OH bond dissociation and the more important is its role in the antioxidant reactivity. The PC-ET mechanism [Eq. (1)] depends as well on the reactivity of the antioxidant with the free radical R^•^ (*i.e.*, DPPH, CH_3_OO^•^). The reaction is thermodynamically favorable (exergonic) if its Gibbs free energy (ΔG) is negative. The PC-ET mechanism can be followed by a second hydrogen atom transfer from the phenoxyl radical (*i.e.*, radical obtained from the first hydrogen atom transfer). Double bond dissociation enthalpy (BDE_d_) becomes an important parameter when calculated BDEs are similar. BDE_d_ are calculated by the following formula:
BDE_d_ = H([ArO^•^− H], 298K) + H(H^•^, 298K) − H(ArO^•^, 298K), (2)
  where H ([ArO^•^− H], 298K) and H (ArO^•^, 298K) are the enthalpies of phenoxyl radical obtained after the first and second hydrogen atom transfer, respectively. As for BDE, the lower is the BDE_d_, the easier is the O-H bond rupture of phenoxyl radical, and the more important is its role in the antioxidant reactivity. We have demonstrated in a previous study the significant role of BDE_d_ parameter to rationalize the antioxidant activity of a series of guaiacol oligomers [11].
(ii) *Electron Transfer-Proton Transfer (ET-PT)*
ArOH + R^•^ → ArOH^+•^+ R^−^ → ArO^•^ + RH
  The ET-PT mechanism consists of two steps. In the first step, an electron transfer (ET) from the styrylpyrone to the free radical leads to the formation of radical cation ArOH^+•^. In the second step, a heterolytic O-H bond dissociation of the radical cation (*i.e.*, proton loss) leads to the formation of a phenoxyl radical. This mechanism is governed by the ionization potential (IP) of the radical cation and the Gibbs free energy reaction of the first step. The lower is the IP value, the easier is the electron transfer and the higher is the antioxidant activity.
(iii) *Sequential Proton Loss Electron Transfer (SPLET)*
ArOH → ArO^−^ + H^+^
ArO^−^ + R^•^ → ArO^•^ + R^−^
R^−^ + H^+^ → RH
  The SPLET mechanism consists of three steps. In the first step, a heterolytic bond dissociation of a phenolic hydroxyl group leads to the formation of a phenoxyl anion and the release of a proton. In the second step, an electron transfer from the phenoxyl anion to the free radical leads to the formation of a phenoxyl radical and an anion (R^−^). In the last step, the protonation of R^−^ leads to the formation of RH. This mechanism is strongly favored under alkaline conditions (e.g., high pH), which may help in the proton of the first step [32,33].
(iv) *Adduct formation (AF)*
ArOH + R^•^ → [ArOH-R]^•^ → stable adducts
  The AF mechanism is more specific and is observed between (a) carbon centered radicals and double bonds; or (b) hydroxyl radicals and aromatic rings. Numerous side reactions may occur that lead to stable adducts from [ArOH-R]^•^. In the present study, we focused on the first two mechanisms PC-ET and ET-PT. The identification of the major mechanism was carried out in two stages, which were (i) determination of the most active OH group (*i.e.*, those with low BDEs); and (ii) thermodynamic and kinetic studies of the reactivity of the active OH groups with free radicals (e.g., CH_3_OO^•^ and DPPH). Both mechanisms lead to the formation of the same final products and thus possess the same free Gibbs reaction energy. Consequently, they are a similar from a thermodynamic point of view (ΔG^PC−ET^ = ΔG^ET−PT^). The favored mechanism was determined kinetically by calculating the activation free energies of PC-ET and ET-PT mechanisms (ΔG^#^).

All theoretical calculations (conformational studies, optimization and frequency calculations) were carried out using DFT methods as implemented in Gaussian09 [34]. The hybrid functional B3B86 proved to be the most suitable for structure-antioxidant activity of polyphenols and is deemed particularly adapted for phenol BDEs by giving a high accuracy compared to the experimental values [12]. Based on these studies, we extended the use of B3P86 hybrid functionals to hispidin derivatives **2–6**. To test the basis set effect, we calculated BDEs for hispidin (**2**) and its tautomer isohispidin (**3**) using double and triple basis sets 6-31+G(d,p) and 6-311+G(d,p), respectively. The obtained BDEs exhibited a difference lower than 0.4 kcal/Mol for the active OH group (12-OH) in gas or PCM (Table 1). Such a difference is low enough to be considered as a part of the global error. Consequently, we used double basis set 6-31+G(d, p) for hispidin oligomers **4–6**. The geometries, enthalpies (H), and Gibbs energies (G) of reactants, intermediates, and products were determined at B3P86/6-31+G(d, p) level. 

All ground states were confirmed by vibrational frequency analysis (*i.e.*, no imaginary frequency). The transition states (TSs) were confirmed by the presence of one imaginary frequency, which was assigned to the vibration mode corresponding to the reaction studied (e.g., bond formation, bond breaking). The TS Gibbs free energy allowed us to evaluate the free ΔG^#^. The activation free energies are known to be underestimated by B3P86, whereas the hybrid functional MPWB1K appeared adequate to reproduce ΔG^#^ [35], especially for hydrogen atom transfer reactions [36]. All calculations related to the kinetic study (*i.e.*, reactant, product, and TS energies) were performed using B3P86 and MPWB1K hybrid functionals. The solvent effects (MeOH) were taken into account implicitly using the polarizable continuum model (PCM). In PCM, the substrate is embedded into a cavity surrounded by a dielectric continuum characterized by its dielectric constant (ε_MeOH_ = 32.613). Different PCM models are available. The calculations were performed using the integral equation formalism variant (IEFPCM) as implemented in the Gaussian09 program [34]. The use of an explicit solvent-surrounded OH phenol group had been investigated and PCM was confirmed to give a better description of BDEs [37]. Trouillas *et al.* tested a hybrid model (*i.e.*, one or two water molecules surrounding OH groups + PCM) for quercetin flavonoid. They showed that resulting BDEs were slightly different from those obtained with PCM only [10]. Regarding the kinetic study of the PC-ET mechanism, the determination of rate constants is more reliable than calculation of activation energies (ΔG^#^) only, since the former would take into account the tunneling in hydrogen shift [38,39]. These rate constants were calculated using transition state theory (TST) according to the following Equation (3):

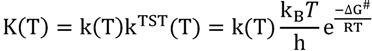
(3)
where k(T) is the transmission coefficient. It represents the quantum tunneling through the reaction barrier. The commonly and easiest used method for evaluating the tunneling is the Wigner method as in Equation (4) below [40]:

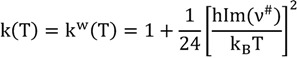
(4)
where ν^#^ is the transition state imaginary frequency corresponding to the reaction coordinate, T the temperature, and h and k_B_ are Planck’s and Boltzmann’s constants, respectively. The imaginary frequency is used to estimate the width of the barrier. However, no term accounts for the barrier height (activation energy) in Winger’s method.

Skodje and Truhlar proposed an improvement over Wigner’s method [41]. The Skodje and Truhlar method accounts for both the barrier width through the imaginary frequency, and the barrier height. The transmission coefficients are obtained by using the following expressions [Equations (5) and (6)]:

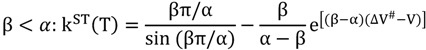
(5)

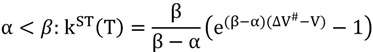
(6)
where:

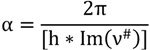
(7)

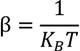
(8)


ΔV^#^ is the zero-point-including potential energy difference between the transition state and the reactants.

## 4. Conclusions

In the present study, we highlighted the role of different descriptors BDEs, BDE_d_, number of OH groups, and conjugated double bond to a catechol group on the antioxidant activity of a series of hispidin oligomers. Theoretical results showed that BDE_d_ is most significant parameter to understand and rationalize the experimental antioxidant activity values of hispidin derivatives. Thermodynamic and kinetic studies showed that the PC-ET mechanism is the favored mechanism for the title compounds to scavenge free radicals.
